# Supplementary figures and images for: SSU1 Checkup, a Rapid Tool for Detecting Chromosomal Rearrangements Related to the SSU1 Promoter in Saccharomyces cerevisiae: An Ecological and Technological Study on Wine Yeast
Source: Front Microbiol. 2020 Jun 29;11:1331. doi: 10.3389/fmicb.2020.01331 (PMC7336578; doi:10.3389/fmicb.2020.01331)

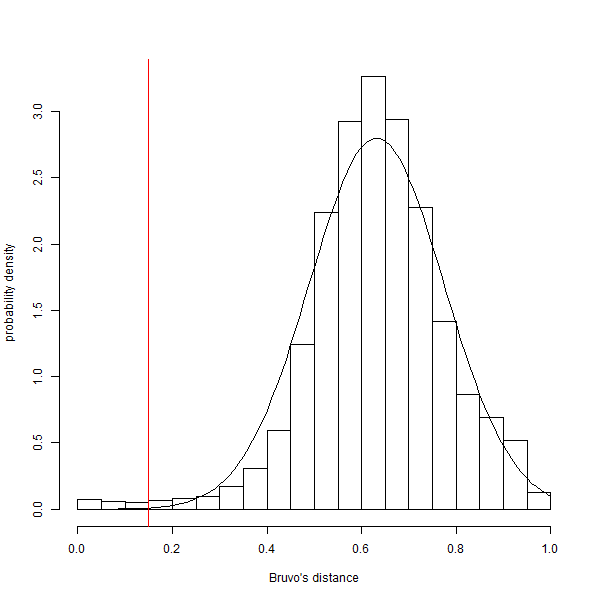

Supplement: FIGURE S1 — Bruvo’s distance distribution and cut off threshold used for removing very similar strains. [file Image_1.tiff]

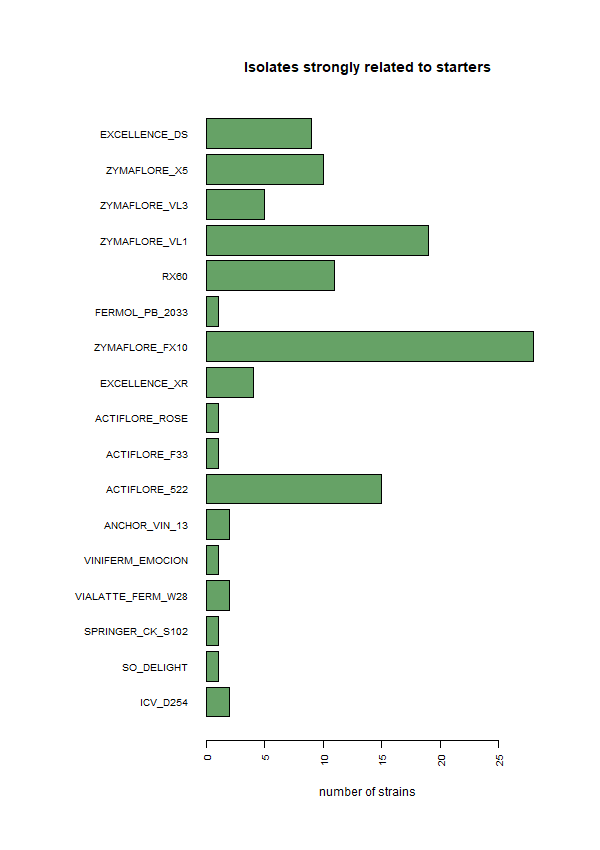

Supplement: FIGURE S2 — Natural isolates closely related to industrial starters. [file Image_2.tiff]

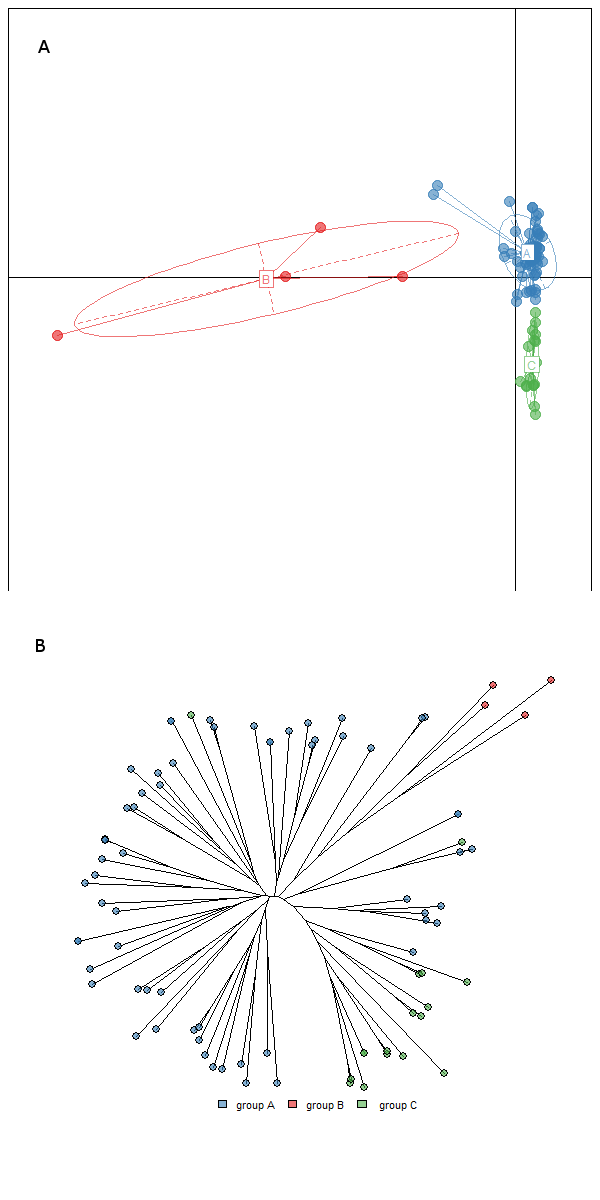

Supplement: FIGURE S3 — (A) Principal Component analysis of 82 commercial starters discriminated by 15 polymorphic loci. The three groups represented (A to C) were inferred by k-mean clustering. The (B) represents the position of the strains according to the inferred groups. [file Image_3.tiff]

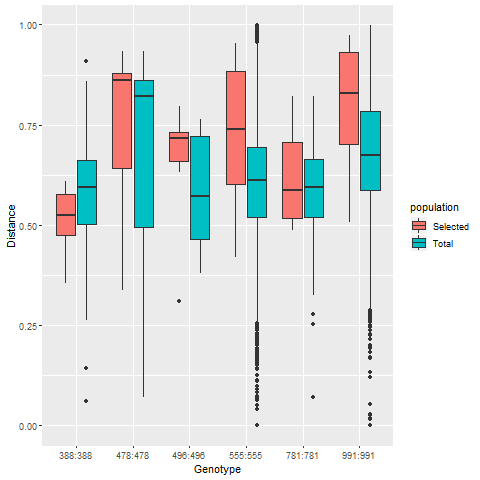

Supplement: FIGURE S5 — Genetic distance between individuals sharing six SSU1 promoter types, only the strains homozygous for these alleles were considered. The color blue and red indicate the distribution of pair wise Bruvo’s genetic distance for the total set of strains and the subset of five strains selected. [file Image_5.tiff]

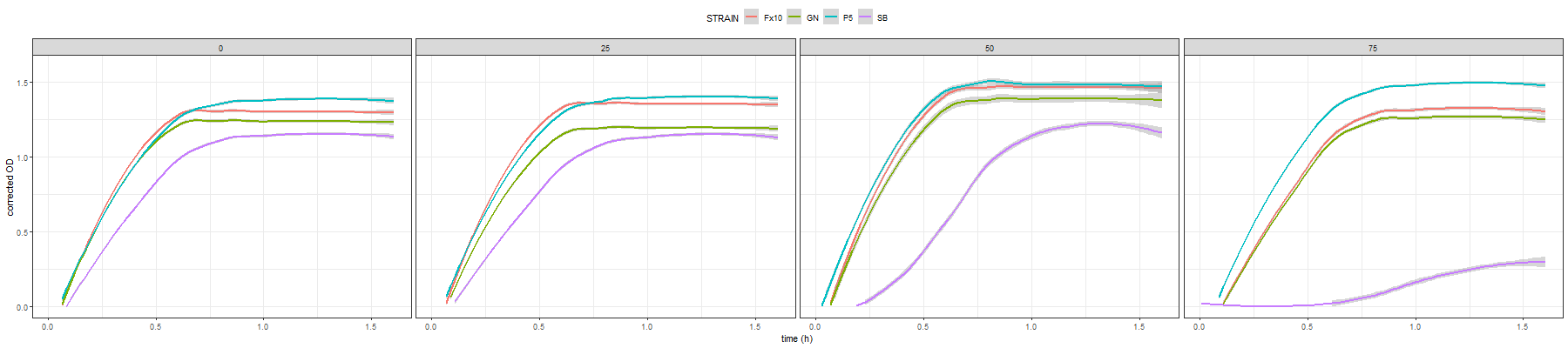

Supplement: FIGURE S6 — Growth curves of reference strains in different grape juice containing different SO2 concentrations expressed in mg/L. The data presented are the average of two independent replicates for the strain Fx10 (red), GN (green), P5 (cyan), and SB (purple). Standard error was figured out by the shaded area. [file Image_6.tiff]
