# Supplementary material for: SSU1 Checkup, a Rapid Tool for Detecting Chromosomal Rearrangements Related to the SSU1 Promoter in Saccharomyces cerevisiae: An Ecological and Technological Study on Wine Yeast
Source: Front Microbiol. 2020 Jun 29;11:1331. doi: 10.3389/fmicb.2020.01331 (PMC7336578; doi:10.3389/fmicb.2020.01331)
Supplement: FIGURE S4 — Schematic representation of a VIII allele of S. cerevisiae. The figure shows the location of the 76 bp Tandem Repeats and the 47 bp Tandem Repeats, as well as the translocation point (TP) and the upstream (5′ region; from the end of the forward primer to the beginning of the 76 bp tandem repeats) and downstream (3′region; from the end of the 47 bp tandem repeats to the translocation point) flanking regions. The hybridization position for the primers forward (F; p1189) and reverse (R; either p1190 or p1194), are indicated. [file Image_4.pdf]

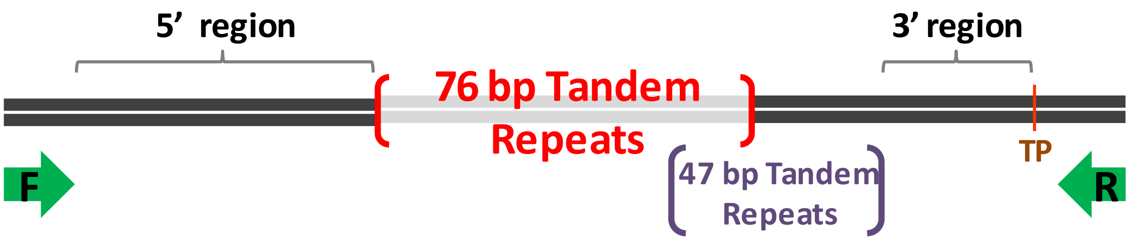

Alleles	Size (bp)	5' region (bp)[1]	Number of Tandem Repeats		3' region (bp)[2]
			76 bp	47 bp	
VIII-t-XVI	388	141	2	1	36
VIII-t-XVI	631	141	5	1	37
VIII-t-XVI	478	141	3	1	37
VIII-t-XVI	555	141	4	1	37
VIII	615	141	1	1	48
VIII	651	141	1	2	37
VIII	649	141	1	2	37
VIII	604	141	1	1	37
VIII	667	141	2	1	37

[1] Length in bp from the end of the forward primer to the beginning of the 76 bp Tandem Repeats.
[4] Length in bp from the end of the 47 bp Tandem Repeats to the translocation point.

Legend to Figure S4.
Schematic representation of a VIII allele of *S. cerevisiae*. The figure shows the location of the 76 bp Tandem(s) and the 47 bp Tandem(s), as well as the translocation point (TP) and the upstream (5'; from the end of the forward primer to the beginning of the 76 bp Tandem) and downstream (3'; from the end of the 47 bp Tandem to the translocation point) flanking regions. The hybridization position for the primers forward (F; p1189) and reverse (R; either p1190 or p1194), are indicated.
